# Timing of puberty in boys and girls: A population‐based study

**DOI:** 10.1111/ppe.12507

**Published:** 2018-10-11

**Authors:** Nis Brix, Andreas Ernst, Lea Lykke Braskhøj Lauridsen, Erik Parner, Henrik Støvring, Jørn Olsen, Tine Brink Henriksen, Cecilia Høst Ramlau‐Hansen

**Affiliations:** ^1^ Department of Public Health, Section for Epidemiology Aarhus University Aarhus Denmark; ^2^ Department of Epidemiology Fielding School of Public Health University of California Los Angeles California USA; ^3^ Department of Public Health, Section for Biostatistics Aarhus University Aarhus Denmark; ^4^ Department of Clinical Epidemiology Aarhus University Hospital Aarhus Denmark; ^5^ Perinatal Epidemiology Research Unit Department of Paediatrics Aarhus University Hospital Aarhus Denmark

**Keywords:** cohort studies, humans, menarche, puberty, sexual development, sexual maturation

## Abstract

**Background:**

A secular trend towards earlier puberty has been observed in girls, while a similar trend has been more uncertain in boys. We estimated current ages at pubertal development in both boys and girls.

**Methods:**

In this population‐based cohort study, 14 759 of 22 439 invited boys and girls born from 2000 to 2003 in the Danish National Birth Cohort gave half‐yearly self‐reported information on puberty from the age of 11.5 years and throughout puberty. This late start of follow‐up limits the estimation of age at onset of puberty but not later pubertal milestones. We estimated mean age at attaining the following pubertal milestones in years with 95% confidence intervals (CI): age at menarche, voice break, first ejaculation of semen and Tanner stages for pubic hair development and breast development or genital development. Further, the difference in mean age at menarche between mothers and daughters was estimated.

**Results:**

In boys, voice break occurred at 13.1 (95% CI 13.0, 13.1) years, first ejaculation of semen occurred at 13.4 (95% CI 13.3, 13.4) years, and Tanner Genital Stage 5 occurred at 15.6 (95% CI 15.5, 15.6) years. In girls, age at menarche occurred at 13.0 (95% CI 13.0, 13.1) years and Tanner Breast Stage 5 occurred at 15.8 (95% CI 15.7, 15.9) years. Daughters had menarche 3.6 (95% CI 3.1, 4.2) months earlier than their mothers had.

**Conclusion:**

These data indicate that age at menarche has declined and to some extent support a decline in age at attaining other markers of pubertal development among boys.

## 1 INTRODUCTION

Existing data show that the age at menarche, a marker of pubertal development in girls, has declined over the last century in Europe and the US, but it remains unsettled whether the decline is continuing or has stopped.^1^, ^2^ A decline in pubertal age in boys has also been suggested, but this is still not investigated in detail.^2^ The reasons for a decline are unknown but is expected to be related to environmental and social factors as the genetic pool remains relatively stable over time. Childhood obesity, improved health, endocrine disrupting chemicals and prenatal exposures have been studied as potential causal factors.^3^, ^4^, ^5^, ^6^ Earlier timing of puberty is a potential concern^2^ as it has been suggested as a risk indicator for some adult diseases, such as obesity, diabetes, heart diseases, breast cancer, and testicular cancer.^7^, ^8^, ^9^, ^10^

In this longitudinal, population‐based cohort study, we estimate the current age at attaining various pubertal milestones in Danish boys and girls, using half‐yearly self‐reported information on pubertal development. Further, we investigate whether daughters had earlier age at menarche than their mothers as an indicator of a potential decline in age at menarche in girls.

## 2 METHODS

### 2.1 Study population

This study was based on the Puberty Cohort, nested within the Danish National Birth Cohort (DNBC). The DNBC includes approximately 92 000 pregnant women and their children and is described in detail elsewhere.^11^ In short, women were recruited during their first trimester and gave information in four computer‐assisted telephone interviews at around 17 and 32 weeks of gestation as well as 6 and 18 months post‐partum. Moreover, the mothers filled in questionnaires when the children were 7 and 11 years of age. Children eligible for being sampled to the Puberty Cohort (n = 56 641) were born during 2000 through 2003 by mothers who participated in the first telephone interview in the DNBC and had not withdrawn their consent by May 2012. Of the 56 641 eligible children, we sampled 22 439 children and only these were invited for participation in the Puberty Cohort. These children were invited to provide half‐yearly information on puberty through Web‐based questionnaires from the age of 11.5 years to 18 years or full maturity, whichever came first. Full maturity was defined as Tanner Stage 5 for genital and pubic hair development in boys and Tanner Stage 5 for breast and pubic hair development in girls.^12^, ^13^ During the study period from August 2012 through March 2017, a total of 14 759 children (participation rate 66%) replied to at least one questionnaire on their current pubertal developmental stage. In total, 73 160 questionnaires were returned (Figure S1). At the end of follow‐up in March 2017, the children were between 14 and 17 years of age.

### 2.2 Timing of puberty in boys and girls

We used a translated version of the growing and changing questionnaire from the British Avon Longitudinal Study of Parents and Children^14^ and drawings of Tanner stages to collect information on puberty. Our questionnaire included Tanner stages for pubic hair (Tanner PH1‐5) and breast (Tanner B1‐5) or genital development (Tanner G1‐5), axillary hair (yes, no), acne (yes, no), menarche (yes, no; age: year and month), first ejaculation of semen (yes, no; age: year and month), and voice break (yes—sometimes, yes—definitive changes, no, don't know). The questionnaire is available in Danish at www.dnbc.dk.

As the information on the pubertal milestones was collected half‐yearly, the pubertal milestone occurred in an interval between an upper and a lower limit. The lower limit was the age at the last questionnaire where the child had not attained the pubertal milestone yet. Similarly, the upper limit was the first time the child had attained the pubertal milestone. The outcome was left‐censored when a milestone was already attained by the first questionnaire, interval‐censored when the pubertal milestone was attained between two questionnaires, and right‐censored when the milestone was not attainted by the last completed questionnaire. When the child reported a specific age (years and months) for first ejaculation or menarche, this age was used as both upper and lower limits. Table S1 gives an overview of the censoring of data.

### 2.3 Maternal age at menarche

In the DNBC's first interview during pregnancy, the women, who were on average 30 years of age, recalled their age (in whole years) at their first menstrual period. Of the 7638 daughters that provided information on age at menarche, 7088 (93%) of the mothers had provided age at menarche.

### 2.4 Background characteristics

We obtained self‐reported information on maternal pre‐pregnancy body mass index (BMI) and maternal smoking in first trimester from the first interview in the DNBC. We obtained information on maternal age at delivery, parity, and birthweight from the Danish Medical Birth Registry and the highest social class of parents from Statistics Denmark. Social class was based on the International Standard Class of Occupation and Education codes (ISCO‐88 and ISCED). Height and weight at 7 years were obtained from the 7‐year follow‐up in the DNBC. Age, height and weight at entry into the Puberty Cohort were obtained from the first returned questionnaire in the Puberty Cohort.

### 2.5 Statistical analysis

The following sampling strategy was employed in the Puberty Cohort. In total, 22 439 children were oversampled from 28 sampling frames: one sampling frame was the 56 641 eligible children from which we randomly sampled 8000 children, and the remaining 27 sampling frames were subgroups of 12 prenatal exposures believed to be important predictors of pubertal timing such as maternal smoking and alcohol consumption during pregnancy, pre‐pregnancy BMI, birthweight, and diabetes mellitus. Hence, sampling weights were applied in all analyses. These sampling weights were the inverse of the probability of being sampled and were calculated based on the sampling fractions of the 28 sampling frames. All models were fitted with robust standard errors to account for the weighing approach for sampling and clustering of siblings.

Analyses were performed in STATA 13.1 MP software (StataCorp, College Station, TX) and R (×64 3.3.1). We estimated age in years at attaining the following pubertal milestones: Tanner Stage 2 to 5, acne and axillary hair in both boys and girls; menarche in girls; and first ejaculation and voice break in boys. To estimate the age at attaining the pubertal milestones in boys and girls, we used a parametric survival analysis for interval‐censored data based on the normal distribution fitted by maximum‐likelihood estimation.^15^ The nonparametric distributions (plotted as the cumulative incidence function) of the interval‐censored pubertal milestones based on the Turnbull Estimator were visually compared with the normal, Weibull, log‐logistic, and gamma distributions.^16^, ^17^ The normal distribution had the best fit (Figures S2 and S3). All pubertal milestones were analysed separately in a univariate model without explanatory variables. Results were presented graphically as cumulative incidence functions and in tabular format as mean age with both 95% confidence interval (CI) and 95% prediction interval (PI). The latter estimates the interval where 95% of participants attained the given pubertal milestone.

Difference in age at menarche between mother and daughter was estimated as follows. In 7088 mother‐daughter pairs with information on age at menarche in both mother and daughter, the mean age at menarche was estimated for mothers by univariate linear regression and then estimated for daughters by the interval‐censored regression model. Then, we calculated the difference of the means between mothers and daughters and 95% CIs were computed using a nonparametric bootstrap procedure with 10 000 replications and normal‐based standard error.

Two sub‐analyses were performed for the analysis on age at attaining the pubertal milestones. In the first sub‐analysis, we excluded all participants with inconsistent reporting of Tanner stages, that is reporting a lower Tanner stage in one questionnaire than in any previous questionnaires. In total, 6572 (86%) girls had consistent information on all Tanner Breast stages, 6219 (81%) girls had consistent information on all Tanner Pubic Hair stages, 5504 (77%) boys had consistent information on all Tanner Genital stages, and 6158 (87%) boys had consistent information on all Tanner Pubic Hair stages. In the second sub‐analysis, we assessed the potential impact of selection bias due to non‐participation (34%) using inverse probability weights.^18^ This was performed by fitting a multivariable logistic regression model for participation using the following explanatory variables: the highest social status of parents, maternal pre‐pregnancy BMI, maternal smoking in first trimester, maternal age at delivery, and parity with continuous covariates (maternal pre‐pregnancy BMI and age at delivery) included as second‐order polynomials. The inverse of the predicted values was used as selection weights. These selection weights were multiplied by the sampling weights from the design of the Puberty Cohort to compute the final weights used in this sub‐analysis.

To assess the sensitivity of violations of the normality assumption, we conducted a simulation study by simulating data with varying degrees of skewness and censoring and assessed the performance of the interval‐censored regression model used in this study (detailed description in Appendix S1, Tables S2 and S3, and Figures S2 through S7).

### 2.6 Ethics

The Committee for Biomedical Research Ethics in Denmark approved the collection of data in the DNBC. A written informed consent was obtained from mothers upon recruitment covering both mother's and offspring's participation until they turn 18 years of age. This study was approved by the Danish Data Protection Agency and the Steering Committee of the DNBC.

## 3 RESULTS

### 3.1 Background characteristics

Table 1 shows background characteristics for 7104 boys and 7655 girls in the Puberty Cohort.

**Table 1:** Background characteristics of 7104 boys and 7655 girls in the Puberty Cohort, Denmark, 2012‐2017

Background Characteristics	Sex		Missing No. (%)
	Boys (n = 7104) No. (%)	Girls (n = 7655) No. (%)	
Maternal characteristics			
Highest social class of parents			30 (0.2)
High grade professional	1686 (23.8)	1761 (23.0)	
Low grade professional	2328 (32.9)	2544 (33.3)	
Skilled worker	1953 (27.6)	2112 (27.6)	
Unskilled worker	942 (13.3)	1032 (13.5)	
Student	137 (1.9)	150 (2.0)	
Economically inactive	37 (0.5)	47 (0.6)	
Maternal age at delivery, years^a^	30.3 (27.6**,** 33.6)	30.4 (27.6, 33.6)	6 (0.0)
Pre‐pregnancy BMI^a^	22.8 (20.6, 25.8)	22.9 (20.7, 26.0)	208 (1.4)
Daily number of cigarettes in 1st trimester			46 (0.3)
Non‐smoker	5142 (72.6)	5457 (71.5)	
−10 cigarettes/d	1505 (21.2)	1647 (21.6)	
>10 cigarettes/d	437 (6.2)	525 (6.9)	
Parity			0 (0.0)
1st child	3647 (51.3)	3816 (49.8)	
2nd child	2364 (33.3)	2671 (34.9)	
3rd or more child	1093 (15.4)	1168 (15.3)	
Child characteristics			
Birthweight in grams^a^	3600 (3230, 4000)	3500 (3145, 3850)	55 (0.4)
Height in centimetres at 7 y^a^	126 (122, 130)	125 (121, 129)	4192 (28.4)
Weight in kilograms at 7 y^a^	24.6 (22.5, 27.0)	24.0 (21.8, 26.4)	4317 (29.2)
BMI at 7 y^a^	15.5 (14.6, 16.5)	15.3 (14.4, 16.5)	4358 (29.5)
Entry into the Puberty Cohort			
Age in years^a^	12.0 (11.6, 12.5)	12.0 (11.5, 12.5)	0 (0.0)
Height in centimetres^a^	155 (149, 160)	155 (150, 161)	11 (0.1)
Weight in kilograms^a^	42.0 (37.0, 49.0)	43.0 (37.0, 49.0)	27 (0.2)
BMI^a^	17.6 (16.2, 19.4)	17.6 (16.0, 19.6)	31 (0.2)

BMI, body mass index; d, day; y, years.

a Presented as median (25 percentile, 75 percentile).

### 3.2 Timing of puberty in boys and girls

**Figure 1:**
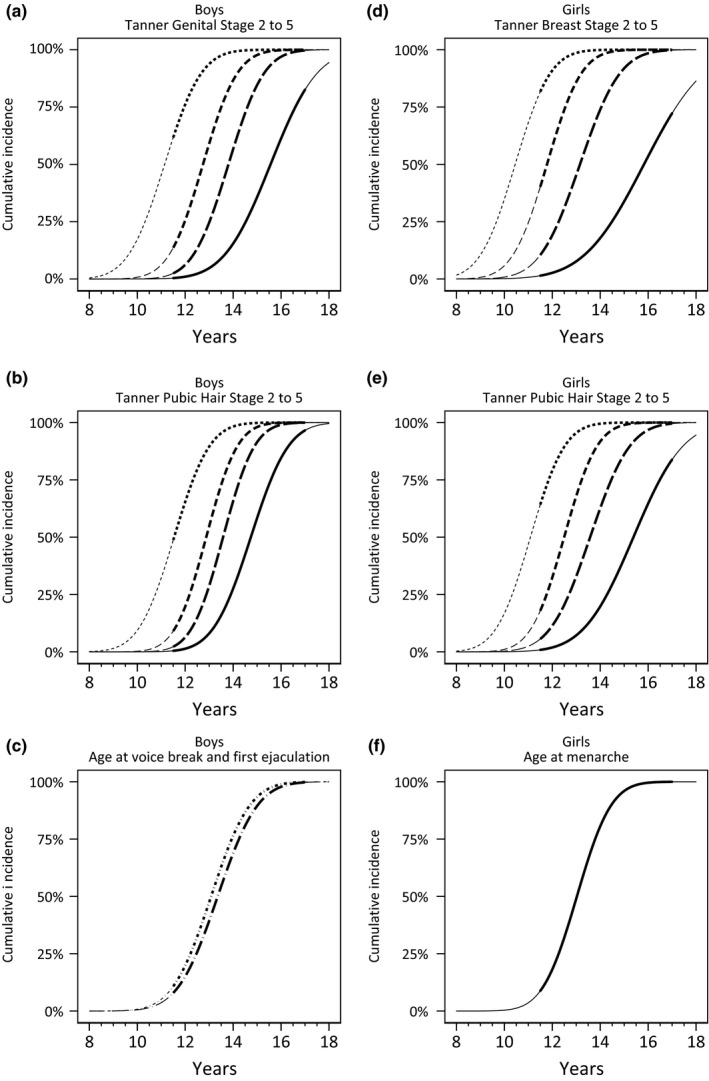
Cumulative incidence of pubertal milestones in 7104 boys and 7655 girls, the Puberty Cohort, Denmark, 2012‐2017. Tanner Stage 2 (short‐dashed line), Tanner Stage 3 (medium‐dashed line), Tanner Stage 4 (long‐dashed line), Tanner Stage 5 (solid line), voice break (short‐dashed‐dotted line), first ejaculation (long‐dashed‐dotted line) and menarche (solid line). Thick lines indicate age range of observed data

Figure 1 shows the cumulative incidence of the age at attaining Tanner stages, voice break and first ejaculation in boys, and Tanner stages and menarche in girls. Table 2 shows the average age at attaining the pubertal milestones with 95% CI and 95% PI. Results remained unchanged when excluding participants with inconsistent reporting of Tanner stages (Table 3) and when using selection weights to account for potential selection bias (Table 4).

**Table 2:** Age at attaining various pubertal milestones for 7104 boys and 7655 girls, the Puberty Cohort, Denmark, 2012‐2017

Pubertal milestones	No. of persons	Age in years		
		Estimate	(95% CI)	(95% PI)
Boys				
Tanner Genital stage 2	7033	11.1	(11.1, 11.2)	(8.8, 13.5)
Tanner Genital stage 3	7034	12.7	(12.7, 12.8)	(10.5, 15.0)
Tanner Genital stage 4	7066	13.8	(13.8, 13.8)	(11.5, 16.1)
Tanner Genital stage 5	7074	15.6	(15.5, 15.6)	(12.6, 18.6)
Tanner Pubic Hair stage 2	7047	11.5	(11.5, 11.6)	(9.3, 13.8)
Tanner Pubic Hair stage 3	7079	12.9	(12.8, 12.9)	(10.9, 14.9)
Tanner Pubic Hair stage 4	7084	13.6	(13.5, 13.6)	(11.5, 15.6)
Tanner Pubic Hair stage 5	7084	14.7	(14.7, 14.8)	(12.3, 17.2)
Axillary hair	7100	13.3	(13.3, 13.4)	(10.6, 16.0)
Acne	7100	12.3	(12.2, 12.3)	(9.7, 14.9)
Voice break	6934	13.1	(13.0, 13.1)	(10.6, 15.6)
First ejaculation	7080	13.4	(13.3, 13.4)	(10.8, 15.9)
Girls				
Tanner Breast stage 2	7634	10.5	(10.3, 10.6)	(8.2, 12.7)
Tanner Breast stage 3	7627	11.8	(11.7, 11.8)	(9.6, 14.0)
Tanner Breast stage 4	7606	13.2	(13.1, 13.2)	(10.6, 15.7)
Tanner Breast stage 5	7635	15.8	(15.7, 15.9)	(11.9, 19.7)
Tanner Pubic Hair stage 2	7596	11.1	(11.0, 11.2)	(8.9, 13.3)
Tanner Pubic Hair stage 3	7590	12.5	(12.5, 12.5)	(10.4, 14.6)
Tanner Pubic Hair stage 4	7605	13.6	(13.5, 13.6)	(11.0, 16.1)
Tanner Pubic Hair stage 5	7634	15.4	(15.3, 15.5)	(12.2, 18.6)
Axillary hair	7651	12.0	(11.9, 12.0)	(9.2, 14.7)
Acne	7651	11.4	(11.4, 11.5)	(8.4, 14.4)
Menarche	7638	13.0	(13.0, 13.1)	(10.8, 15.2)

CI, confidence interval; PI, prediction interval.

**Table 3:** Age at attaining various pubertal milestones when excluding inconsistencies on Tanner stages, the Puberty Cohort, Denmark, 2012‐2017

Pubertal milestones	No. of persons	Age in years		
		Estimate	(95% CI)	(95% PI)
Boys				
Tanner Genital stage 2	5504	11.1	(11.1, 11.2)	(8.7, 13.6)
Tanner Genital stage 3	5504	12.7	(12.6, 12.7)	(10.5, 14.8)
Tanner Genital stage 4	5504	13.6	(13.5, 13.6)	(11.4, 15.7)
Tanner Genital stage 5	5504	15.2	(15.1, 15.3)	(12.4, 18.1)
Tanner Pubic Hair stage 2	6158	11.5	(11.5, 11.6)	(9.2, 13.9)
Tanner Pubic Hair stage 3	6158	12.8	(12.8, 12.9)	(10.9, 14.8)
Tanner Pubic Hair stage 4	6158	13.5	(13.4, 13.5)	(11.5, 15.4)
Tanner Pubic Hair stage 5	6158	14.6	(14.5, 14.6)	(12.2, 17.0)
Girls				
Tanner Breast stage 2	6572	10.4	(10.3, 10.6)	(8.1, 12.8)
Tanner Breast stage 3	6572	11.8	(11.7, 11.8)	(9.6, 14.0)
Tanner Breast stage 4	6572	13.0	(13.0, 13.1)	(10.5, 15.6)
Tanner Breast stage 5	6572	15.5	(15.5, 15.6)	(11.8, 19.3)
Tanner Pubic Hair stage 2	6219	11.1	(11.0, 11.2)	(8.8, 13.4)
Tanner Pubic Hair stage 3	6219	12.5	(12.4, 12.5)	(10.3, 14.6)
Tanner Pubic Hair stage 4	6219	13.4	(13.4, 13.5)	(10.9, 16.0)
Tanner Pubic Hair stage 5	6219	15.1	(15.0, 15.2)	(12.0, 18.2)

CI, confidence interval; PI, prediction interval.

**Table 4:** Age at attaining various pubertal milestones in years using selection weights^a^ for 7104 boys and 7655 girls, the Puberty Cohort, Denmark, 2012‐2017

Pubertal milestones	No. of persons^b^	Age in years		
		Estimate	(95% CI)	(95% PI)
Boys				
Tanner Genital stage 2	6893	11.1	(11.1, 11.2)	(8.8, 13.5)
Tanner Genital stage 3	6896	12.7	(12.7, 12.8)	(10.5, 14.9)
Tanner Genital stage 4	6926	13.8	(13.7, 13.8)	(11.5, 16.1)
Tanner Genital stage 5	6935	15.5	(15.5, 15.6)	(12.5, 18.6)
Tanner Pubic Hair stage 2	6907	11.5	(11.5, 11.6)	(9.3, 13.8)
Tanner Pubic Hair stage 3	6939	12.9	(12.8, 12.9)	(10.9, 14.9)
Tanner Pubic Hair stage 4	6944	13.6	(13.5, 13.6)	(11.5, 15.6)
Tanner Pubic Hair stage 5	6944	14.7	(14.7, 14.8)	(12.3, 17.2)
Axillary hair	6958	13.3	(13.3, 13.4)	(10.7, 16.0)
Acne	6958	12.3	(12.2, 12.3)	(9.7, 14.8)
Voice break	6796	13.1	(13.0, 13.1)	(10.6, 15.6)
First ejaculation	6940	13.3	(13.3, 13.4)	(10.8, 15.9)
Girls				
Tanner Breast stage 2	7486	10.5	(10.3, 10.6)	(8.2, 12.7)
Tanner Breast stage 3	7479	11.8	(11.7, 11.8)	(9.5, 14.0)
Tanner Breast stage 4	7458	13.1	(13.1, 13.2)	(10.6, 15.7)
Tanner Breast stage 5	7487	15.8	(15.7, 15.9)	(11.9, 19.7)
Tanner Pubic Hair stage 2	7448	11.1	(11.0, 11.2)	(8.9, 13.3)
Tanner Pubic Hair stage 3	7443	12.5	(12.5, 12.5)	(10.4, 14.6)
Tanner Pubic Hair stage 4	7459	13.6	(13.5, 13.6)	(11.0, 16.2)
Tanner Pubic Hair stage 5	7486	15.4	(15.3, 15.5)	(12.2, 18.6)
Axillary hair	7503	12.0	(11.9, 12.0)	(9.2, 14.7)
Acne	7503	11.4	(11.4, 11.5)	(8.4, 14.4)
Menarche	7490	13.0	(13.0, 13.0)	(10.8, 15.2)

CI, confidence interval; PI, prediction interval.

a Selection weights based on the highest social status of parents, maternal pre‐pregnancy body mass index, maternal smoking in first trimester, maternal age at delivery and parity.

b No. of persons in Table 4 is fewer than that in Table 2 due to missing data on covariates for estimation of selection weights.

### 3.3 Simulation study

The simulation study showed that the estimate for the Tanner B2 with 90% left censoring is unbiased if the assumption of normality is correct but may be biased by up to +/−0.35 years under realistic violations of the assumption of normality, whereas estimates for all later milestones were biased with up to +/−0.05 years under the same realistic violations (Table S4).

### 3.4 Difference in age at menarche

Age at menarche occurred 3.6 (−3.6 [95% CI: −4.2, −3.1]) months earlier in daughters than in their mothers. As the mean maternal age at delivery was 30 years, this potential decline in age at menarche occurred over approximately 30 years.

## 4 COMMENT

### 4.1 Principal findings

This longitudinal cohort study provides current estimates for age at attaining various pubertal milestones in both boys and girls. The age at menarche in daughters was estimated to occur 3.6 months earlier than that in their mothers, which indicates that the age at menarche may have declined.

### 4.2 Strengths of the study

The main strengths of this study include a large sample size with longitudinal data collection or short recall periods for age at menarche and first ejaculation, allowing us to estimate both age at attaining the pubertal milestones and difference in age at menarche between mothers and daughters with some precision. The large set of puberty markers allow comparability with both previous and future studies, and the high participation rate reduces the risk of selection bias. Further, our results were robust in sub‐analyses in which we excluded children with inconsistent reporting of Tanner stages and took potential selection bias into account.

### 4.3 Limitations of the data

A limitation of the analysis of age at attaining the pubertal milestone in Danish boys and girls is the uncertainty related to self‐reported information on Tanner stages collected through questionnaires, which is expected to be more prone to misclassification than information collected in clinical examinations.^19^ However, studies using clinical examinations tend to have low participation rates, which may cause selection bias. Using self‐reported information on puberty, we were able to conduct a large, population‐based study with a more representative sample of Danish children, but at the cost of some measurement errors. Another limitation is the late start of follow‐up at the age of 11.5 years, resulting in a high proportion of children that had already attained the earliest pubertal milestones (Tanner stage 2) when the first questionnaire was distributed (90% of girls were Tanner B2+, 78% of girls were Tanner PH2+, 76% of boys were Tanner G2+, and 66% of boys were Tanner PH2+). For the estimates for the earliest milestones to be valid, the assumption of normally distributed residuals is critical. Data related to the later milestones supported this assumption, but assessment of this assumption was impossible for onset of puberty due to the high degree of left censoring. In a simulation study, we mimicked the late start of follow‐up for Tanner B2 under scenarios with both normal and skewed distributions of the residuals. These simulations showed that our estimate for age at Tanner B2 is unbiased if the assumption of normality is correct but may be biased by up to 3‐4 months under realistic violations of the assumption of normality (Table S4). However, our estimates were robust to these violations of the normality assumption when data in the middle of the age distribution were available. Consequently, follow‐up from 11.5 years may result in biased estimates for Tanner Stage 2 if the assumption of normality was not fulfilled, but the potential bias is likely negligible for later pubertal milestones.

The major limitation in the analysis of difference in age at menarche between mothers and daughters was the different methods used for data collection. For daughters, age at menarche was collected during puberty when their menarche occurred, thereby reducing the risk of misclassification, whereas age at menarche for the mothers was obtained during pregnancy when they were around 30 years, that is 16.4 (25 and 75 percentiles: 13.5, 19.8) years after their menarche. If mothers tend to systematically report an older age at menarche than their actual age, this misclassification could explain the observed difference. However, recalled age at menarche has been found to be remembered even in middle‐aged women with some accuracy and without any systematic over‐reporting or under‐reporting, indicating that our estimate need not be biased in one direction by recalled age at menarche.^20^

### 4.4 Interpretation

With regard to boys in this study, voice break occurred at an average age of 13.1 years, which is considerably earlier than former studies reporting between 15.5 and 14.0 years in the period 1968 to 2005.^21^, ^22^, ^23^ However, the reporting of these ages may not be directly comparable as those studies used either an evaluator to assess whether the boys had voice break during speaking^21^ or singing^23^ or the boys recalled timing of voice break during adulthood.^22^ Age at first ejaculation also occurred more than 1 year earlier in our study than an earlier study from 2005 using recalled age at first ejaculation (13.4 vs 14.7 year, respectively).^22^ Tanner G2 and PH2 occurred around half a year earlier than most recent Danish data, whereas the age at Tanner G5 and PH5 were similar.^24^, ^25^ However, these results are not directly comparable as the former studies used clinical examination with low participation rates of 25%‐35%, implying an increased risk of selection bias, but probably with less misclassified information on the pubertal milestones than in our study, especially for Tanner Stage 2.^24^, ^25^ Even though differences in design across studies could play a major role in the observed difference in ages, our results point towards earlier timing of puberty for some milestones in Danish boys. This is in line with earlier studies that have indicated a secular decline towards earlier timing of puberty in Danish boys: one Danish study found a 0.4‐year decline in onset of the growth spurt and 0.3‐year decline in age at peak height velocity over a 40‐year period from 1930 to 1969 in the Municipality of Copenhagen, Denmark,^26^ another study found a 4‐month decline in age at voice break over a 10‐year period from 1994 to 2003 in Danish choir boys;^23^ and a third study found a 3‐month decline in age at onset of puberty, measured by testicular volume >3 mL, over a 15‐year interval from the period 1991‐1993 to the period 2006‐2008.^25^

Girls in this study had menarche at the age of 13.0, which is similar to recent data from the Copenhagen Puberty Study.^27^ These data are probably comparable with ours as both were self‐reported as either current status or specific age with short recall, but there may be differences at the population level due to participation (35% and 66%, respectively). Our data indicated a decline in age at menarche of 3.6 months when comparing mothers with daughters. We speculate that this potential decline might be larger owing to the following reporting pattern. A mother may likely report an age at menarche of, for example, 13 years because her menarche occurred during that period of life where she refers to her age as 13 years and hence somewhere in the range 13.00 to 13.99 years and on average closer to 13.5 than 13 years. If mothers in fact reported after this pattern, the average maternal age at menarche might be up to 6 months older and the decline might, therefore, be up to 6 months larger. For this reason, the estimated decline in age at menarche may be a conservative estimate. The age at Tanner B2 and PH2 were slightly higher than the age found in most recent Danish data, but our estimates may be prone to bias due to the high degree of left censoring.^27^ Later stages, such as Tanner B4 and PH4, were up to 1 year later than shown in most recent Danish studies based on data from 2006 to 2008, but comparable to data from 1991 to 1993.^27^ These differences may be attributed to differences in study design, study populations and reporting errors. A decline in pubertal age has been observed in other Danish studies: the age at breast development declined by 1.0 year and age at menarche declined by 0.3 year over a 15‐year period in the Copenhagen Puberty Study described above^27^ and the onset of the growth spurt declined 0.2 years and the age at peak height velocity declined 0.5 years over a 40‐year period from 1930 to 1969 in girls in primary school from the Municipality of Copenhagen.^26^

The current data may be suitable to evaluate the cut‐off ages for delayed puberty, which is usually defined as no sign of genital development in boys and breast development in girls at an age >2 or >2.5 standard deviations (SD) above the population mean, depending on the definition.^28^ Traditionally, the cut‐off ages for delayed puberty have been 14 years in boys and 13 years in girls, which is currently in use in Denmark.^28^ Using data from this study, the cut‐offs for boys (Tanner G2) would be 13.6 or 14.2 years according to the definition of >2.0 SD or >2.5 SD, respectively. The cut‐offs for girls (Tanner B2) would be 12.8 or 13.3 years according to the definition of >2.0 SD or >2.5 SD, respectively. Even though these findings are compatible with current cut‐off ages, we stress the importance of balancing the risk of unnecessary medical examination with the risk of overlooking underlying pathology when choosing cut‐off ages.

A potential decline in pubertal age may be driven by an increased prevalence of childhood obesity.^29^, ^30^ However, the children in this study were on average normal weight with a BMI just below the population mean,^31^ indicating that this is not a likely explanation for a potential decline in our cohort. Changes in exposures to endocrine‐disrupting chemicals provide another promising explanation,^4^ but the effect of such chemicals has proven difficult to study due to, for example, multiple relevant exposure windows and exposure to a mixture of chemicals with potential opposing effect on pubertal development.^5^ Emerging evidence suggests that various exposures during fetal life may also influence timing of puberty,^6^, ^14^, ^32^, ^33^ but whether prenatal factors can explain a secular trend in timing of puberty is unsettled.

A decline in timing of puberty may be of concern as early puberty may be causally related to later diseases in adulthood, such as obesity, diabetes mellitus, cardiovascular diseases, testicular cancer, and breast cancer.^7^, ^8^, ^9^, ^10^ This calls for continued surveillance for secular trends in pubertal timing. This study was aimed to estimate a wide range of pubertal markers as reliable as possible from a nationwide sample. These data were intended to serve as the basis for comparison with both former and future data on timing of puberty.

We only compared our data with former Danish data to increase the comparability as timing of puberty varies throughout the world.^34^ However, a decline in pubertal timing in Denmark may well be generalizable to other countries with similar genetic background and distribution of social and environmental factors.

## 5 CONCLUSIONS

In boys, most pubertal milestones were attained at younger ages than previously reported, but this apparent decline should be evaluated with caution. In girls, age at menarche occurred 3.6 months earlier in daughters than in their mothers.
