# Supplementary figures and images for: Timing of puberty in boys and girls: A population‐based study
Source: Paediatr Perinat Epidemiol. 2018 Oct 11;33(1):70–8. doi: 10.1111/ppe.12507 (PMC6378593; doi:10.1111/ppe.12507)

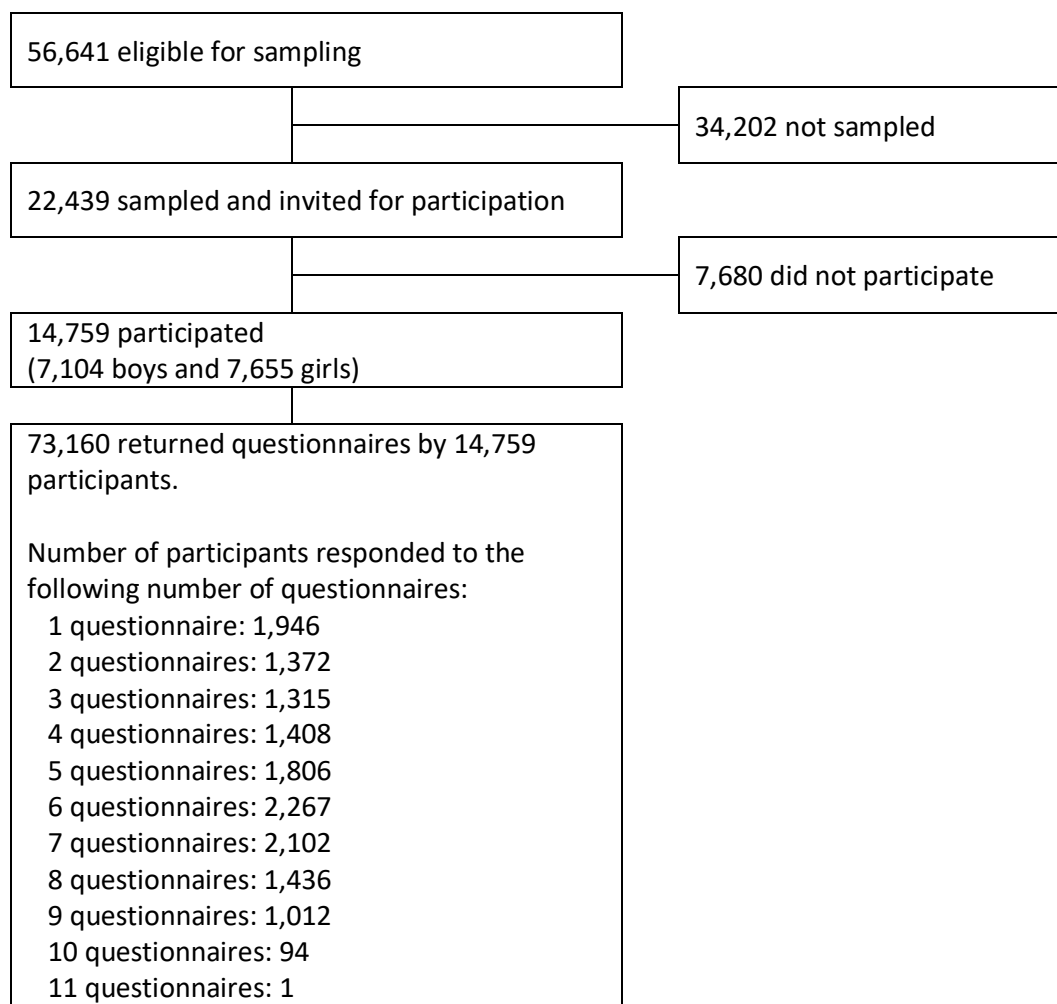

**SUPPLEMENTARY FIGURE 1.** Flow of participation in the Puberty Cohort, Denmark, 2012-2017.

Supplement: Supplementary file 1 [file PPE-33-70-s001.pdf]

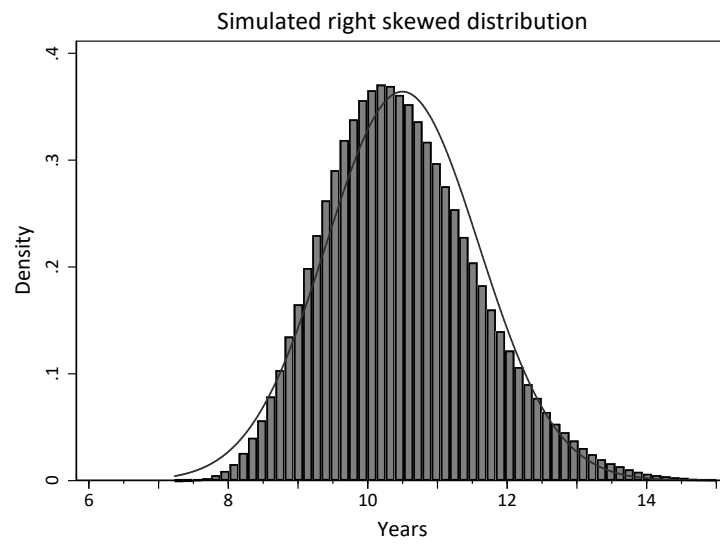

**SUPPLEMENTARY FIGURE 4.** Histogram of simulated right skewed distribution.

Supplement: Supplementary file 4 [file PPE-33-70-s004.pdf]

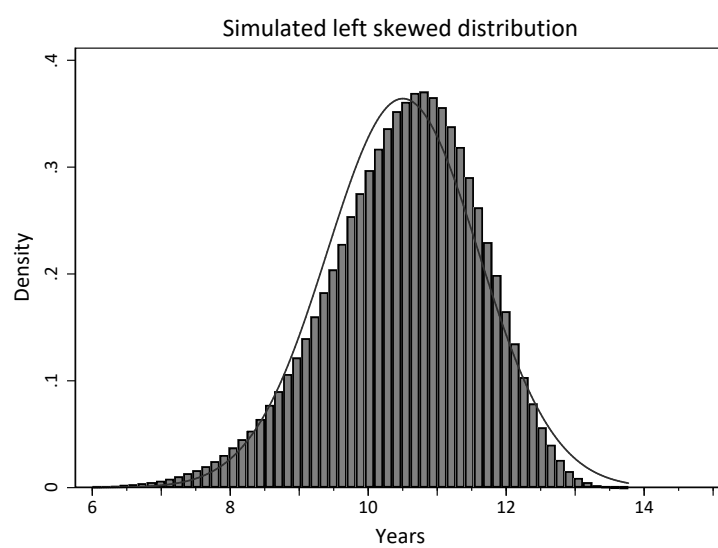

**SUPPLEMENTARY FIGURE 5.** Histogram of simulated left skewed distribution.

Supplement: Supplementary file 5 [file PPE-33-70-s005.pdf]
