# Supplementary material for: Timing of puberty in boys and girls: A population‐based study
Source: Paediatr Perinat Epidemiol. 2018 Oct 11;33(1):70–8. doi: 10.1111/ppe.12507 (PMC6378593; doi:10.1111/ppe.12507)
Supplement: Supplementary file 3 [file PPE-33-70-s003.pdf]

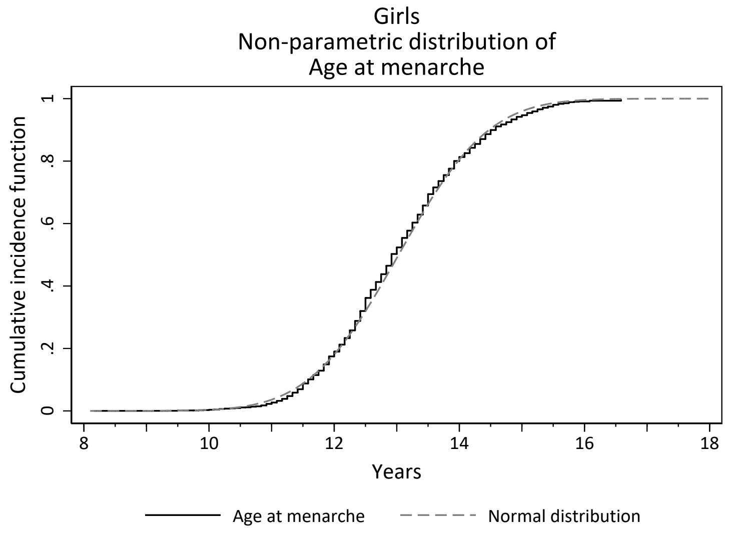

SUPPLEMENTARY FIGURE 3. Cumulative incidence of age at menarche in 7,655 girls, the Puberty Cohort, Denmark, 2012-2017.
