# Supplementary material for: Timing of puberty in boys and girls: A population‐based study
Source: Paediatr Perinat Epidemiol. 2018 Oct 11;33(1):70–8. doi: 10.1111/ppe.12507 (PMC6378593; doi:10.1111/ppe.12507)
Supplement: Supplementary file 6 [file PPE-33-70-s006.pdf]

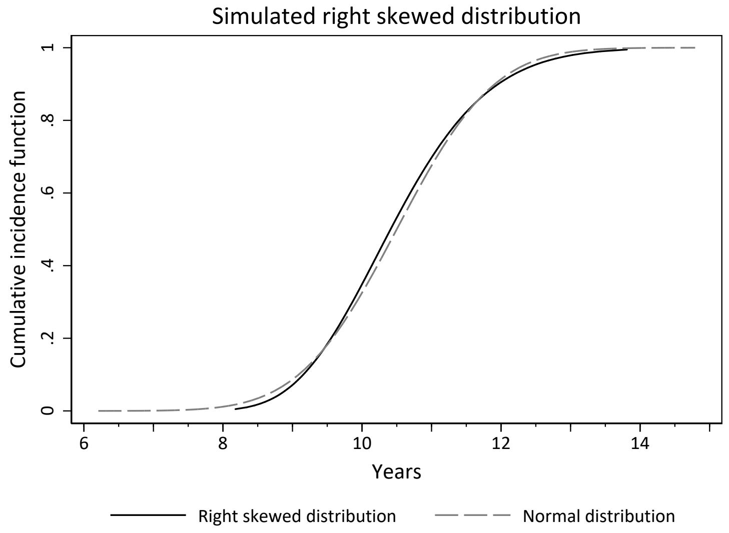

SUPPLEMENTARY FIGURE 6. Cumulative incidence function of simulated right skewed distribution compared to the normal distribution.
