# Supplementary material for: Timing of puberty in boys and girls: A population‐based study
Source: Paediatr Perinat Epidemiol. 2018 Oct 11;33(1):70–8. doi: 10.1111/ppe.12507 (PMC6378593; doi:10.1111/ppe.12507)
Supplement: Supplementary file 8 [file PPE-33-70-s008.pdf]

SUPPLEMENTARY TABLE 1. Censoring of pubertal milestones for 7,104 boys and 7,655 girls, the Puberty Cohort, Denmark, 2012-2017.

Pubertal milestones	Left-censored No. (%)	Uncensored No. (%)	Right-censored No. (%)	Interval-censored No. (%)
Boys				
Tanner Genital Stage 2	5,345 (76.0)	0 (0.0)	357 (5.1)	1,330 (18.9)
Tanner Genital Stage 3	2,073 (29.5)	0 (0.0)	1,492 (21.2)	3,468 (49.3)
Tanner Genital Stage 4	561 (7.9)	0 (0.0)	2,869 (40.6)	3,635 (51.5)
Tanner Genital Stage 5	67 (0.9)	0 (0.0)	5,093 (72.0)	1,913 (27.0)
Tanner Pubic Hair Stage 2	4,627 (65.7)	0 (0.0)	547 (7.8)	1,872 (26.6)
Tanner Pubic Hair Stage 3	1,612 (22.8)	0 (0.0)	1,531 (21.6)	3,935 (55.6)
Tanner Pubic Hair Stage 4	615 (8.7)	0 (0.0)	2,454 (34.6)	4,014 (56.7)
Tanner Pubic Hair Stage 5	117 (1.7)	0 (0.0)	4,128 (58.3)	2,838 (40.1)
Axillary Hair	1,313 (18.5)	0 (0.0)	2,217 (31.2)	3,569 (50.3)
Acne	2,993 (42.2)	0 (0.0)	1,090 (15.4)	3,016 (42.5)
Voice break	1,655 (23.9)	0 (0.0)	1,818 (26.2)	3,460 (49.9)
First ejaculation	3 (0.0)	4,482 (63.3)	2,563 (36.2)	31 (0.4)
Girls				
Tanner Breast Stage 2	6,859 (89.9)	0 (0.0)	89 (1.2)	684 (9.0)
Tanner Breast Stage 3	4,407 (57.8)	0 (0.0)	505 (6.6)	2,713 (35.6)
Tanner Breast Stage 4	1,556 (20.5)	0 (0.0)	1,889 (24.8)	4,159 (54.7)
Tanner Breast Stage 5	173 (2.3)	0 (0.0)	5,203 (68.2)	2,257 (29.6)
Tanner Pubic Hair Stage 2	5,895 (77.6)	0 (0.0)	248 (3.3)	1,451 (19.1)
Tanner Pubic Hair Stage 3	2,582 (34.0)	0 (0.0)	1,099 (14.5)	3,907 (51.5)
Tanner Pubic Hair Stage 4	984 (12.9)	0 (0.0)	2,497 (32.8)	4,122 (54.2)
Tanner Pubic Hair Stage 5	143 (1.9)	0 (0.0)	4,849 (63.5)	2,640 (34.6)
Axillary Hair	3,949 (51.6)	0 (0.0)	737 (9.6)	2,963 (38.7)
Acne	4,886 (63.9)	0 (0.0)	595 (7.8)	2,168 (28.3)
Menarche	1 (0.0)	6,163 (80.7)	1,467 (19.2)	5 (0.1)
