# Supplementary material for: Timing of puberty in boys and girls: A population‐based study
Source: Paediatr Perinat Epidemiol. 2018 Oct 11;33(1):70–8. doi: 10.1111/ppe.12507 (PMC6378593; doi:10.1111/ppe.12507)
Supplement: Supplementary file 9 [file PPE-33-70-s009.pdf]

SUPPLEMENTARY TABLE 2. Mean, median, standard deviation and 90-percentile for the simulated distributions

	Normal distribution (Y_{normal})	Right skewed distribution ($Y_{\mathrm{rightskew}}$)	Left skewed distribution (Y_{leftskew})
Mean, years	10.50	10.50	10.50
Standard deviation, years	1.1	1.1	1.1
Median, years	10.50	10.41[a]	10.59[a]
90-percentile, years	11.91	11.96[a]	11.86[a]

[a]Obtained by simulation.
