# Supplementary material for: Timing of puberty in boys and girls: A population‐based study
Source: Paediatr Perinat Epidemiol. 2018 Oct 11;33(1):70–8. doi: 10.1111/ppe.12507 (PMC6378593; doi:10.1111/ppe.12507)
Supplement: Supplementary file 10 [file PPE-33-70-s010.pdf]

SUPPLEMENTARY TABLE 3. Overview of simulation approach and performance measures

	Simulation experiment 1	Simulation experiment 2	Simulation experiment 3
Simulation approach			
Simulated girls	8,000	8,000	8,000
Replications, S	*S*=10,000	*S*=10,000	*S*=10,000
Distribution(s)	Y_{normal}	$Y_{\text{rightskew}}$ & Y_{leftskew}	$Y_{\text{rightskew}}$ & Y_{leftskew}
First questionnaire, years[a]	90p + N(0; 0.625)	90p + N(0; 0.625)	9.5 + N(0; 0.625)
Subsequent questionnaires, years[b]	|N(0.5; 0.0625)| later	|N(0.5; 0.0625)| later	|N(0.5; 0.0625)| later
Number of questionnaires	12	12	5
Proportion of left censoring	~90%	~90%	15-20%
Proportion of interval censoring	~10%	~10%	60-65%
Proportion of right censoring	~0%	~0%	15-20%
Performance measures			
Estimated mean age at Tanner B2	Yes	Yes	Yes
Bias in mean	Yes	Yes	Yes
Bias in median	-	Yes	Yes
Coverage of true mean	Yes	Yes	Yes
Coverage of true median	-	Yes	Yes

Abbreviations: 90p = 90-percentile.

[a]N(0; 0.625) indicates a random draw from a normal distribution with mean 0 and standard deviation 0.0625.

[b]|N(0.5; 0.625)| indicates the absolute value of a random draw from a normal distribution with mean 0.5 and standard deviation 0.0625.
