# Supplementary material for: Timing of puberty in boys and girls: A population‐based study
Source: Paediatr Perinat Epidemiol. 2018 Oct 11;33(1):70–8. doi: 10.1111/ppe.12507 (PMC6378593; doi:10.1111/ppe.12507)
Supplement: Supplementary file 11 [file PPE-33-70-s011.pdf]

SUPPLEMENTARY TABLE 4. Main results of simulation experiment 1, 2 and 3

	Simulation 1[a]	Simulation 2[b]		Simulation 3[c]	
Performance measures	Normal distribution (Y_{normal})	Right skewed distribution $(Y_{\text{rightskew}})$	Left skewed distribution (Y_{leftskew})	Right skewed distribution $(Y_{\text{rightskew}})$	Left skewed distribution (Y_{leftskew})
Proportion of left censoring	90.0%	90.0%	90.0%	18.5%	17.8%
Proportion of interval censoring	10.0%	10.0%	10.0%	63.7%	63.6%
Proportion of right censoring	0.0%	0.0%	0.0%	17.9%	18.6%
Estimated mean age at Tanner B2, years	10.50	10.15	10.85	10.45	10.55
$\text{Bias}_{\text{mean}}$, years	0.00	-0.35	0.35	-0.05	0.05
$\text{Bias}_{\text{median}}$, years	-	-0.26	0.26	0.04	-0.04
Coverage of true mean	94.8%	0.0%	0.0%	3.8%	3.8%
Coverage of true median	-	2.1%	0.0%	12.2%	11.9%

Abbreviations: Tanner B2, Tanner Breast stage 2.

[a]Simulation experiment 1 with ~90% left-censoring and a normal distribution.

[b]Simulation experiment 2 with ~90% left-censoring and non-normal distributions.

[c]Simulation experiment 3 with ~60-65% left-censoring and non-normal distributions.
