# Supplementary material for: Timing of puberty in boys and girls: A population‐based study
Source: Paediatr Perinat Epidemiol. 2018 Oct 11;33(1):70–8. doi: 10.1111/ppe.12507 (PMC6378593; doi:10.1111/ppe.12507)
Supplement: Supplementary file 12 [file PPE-33-70-s012.docx]

**Supporting Information for:**

**Timing of Puberty in Boys and Girls: A Population-Based Study**

Performance of interval censored regression with substantive left-censoring and skewed distributions: A simulation study

**1 INTRODUCTION**

In the present study, a high proportion of Tanner Breast Stage 2 (Tanner B2) were left censored (~90%) due to late start of follow-up in the Puberty Cohort at around 11.5 years. From a theoretical point of view, the interval censored regression model will yield valid estimates if age at Tanner B2 is normally distributed, but if age at Tanner B2 is not normally distributed, estimates may become biased.

In our empirical data, we visually checked the assumption of normality for each of the pubertal milestones by plotting the cumulative incidence function based on the normal distribution against the non-parametric Turnbull Estimator. This is shown in Supplementary Figure 2 and 3 for breast development (Tanner B2 to B5) and menarche. As the left 90% of Tanner B2 is not observed due to late start of follow-up in the Puberty Cohort, it is not possible to check the normality assumption for this milestone, and we have to rely on extrapolations from later milestones, such as Tanner B3, B4, B5 and menarche. Using simulations, we aimed to explore the following:

- Simulation 1: Magnitude and direction of bias if 90% of observations were left-censored and normally distributed.
- Simulation 2: Magnitude and direction of bias if 90% of observations were left-censored but not normally distributed.
- Simulation 3: Magnitude and direction of bias if 60-65% of observations were interval-censored in the middle of the age distribution but not normally distributed.

**2 METHODS**

**2.1 Simulation of normal, right skewed and left skewed distributions**

In the Puberty Cohort, age at attaining Tanner B2 was estimated to occur at 10.5 years with a standard deviation of 1.1 years. Thus, we simulated a normal distribution ($Y_{\text{normal}}$), a right skewed distribution ($Y_{\mathrm{rightskew}}$), and a left skewed distribution ($Y_{\mathrm{leftskew}}$) to have exactly mean 10.5 years and standard deviation 1.1 years.

The normal distribution ($Y_{\text{normal}}$) was simulated as follows:

$$Y_{\text{normal}} \sim N(10.5;1.1)$$

To simulate the right and left skewed distributions ($Y_{\mathrm{rightskew}}$ and $Y_{\mathrm{leftskew}}$), we first simulated standardized right and left skewed distributions ($Y_{standard,rightskew}$ and $Y_{standard,leftskew}$) with mean 0 and standard deviation of 1 using the method developed for simulating non-normal distributions described by Fleishman 1978:^1^

$X= a + b\cdot Z+c\cdot Z^{2}$, where $Z\sim N(0;1)$

The mean of the simulated distribution is 0 if $a=-c$,^1^ and the variance of X, $\mathrm{var}\left( X \right)$, is given by:^1^

$$\mathrm{var}\left( X \right)= b^{2}+2{\cdot c}^{2}$$

Thus, we can create a standardized skewed distribution ($X_{\text{standard}}$) with mean 0 and standard deviation 1:

$X_{\text{standard}}=\frac{a + b\cdot Z+c\cdot Z^{2}}{\sqrt{b^{2}+2{\cdot c}^{2}}}$, where $Z\sim N(0;1)$ and $a=-c$

We chose the values a = -0.08, b = 1.0, and c = 0.08 to create a realistic scenario of skewness as observed for later milestones in our empirical data, e.g., age at menarche. Thus, we created a standardized right skewed distribution ($Y_{standard,rightskew}$):

Step 1a: $Y_{standard,rightskew}=\frac{-0.08 + 1.0\cdot Z+0.08\cdot Z^{2}}{\sqrt{{1.0}^{2}+2{\cdot0.08}^{2}}}$, where $Z\sim N(0;1)$

The standardized left skewed distribution ($Y_{standard,leftskew}$) was created by mirroring the right skewed:

Step 1b: $Y_{standard,leftskew}={-Y}_{standard,rightskew}$

Then, we created the right and left skewed distributions by multiplying with the standard deviation for Tanner B2 and adding the mean age at attaining Tanner B2:

Step 2a: $Y_{\mathrm{rightskew}}=Y_{standard,rightskew}\cdot1.1+10.5$

Step 2b: $Y_{\mathrm{leftskew}}=Y_{standard,leftskew}\cdot1.1+10.5$

Hence, we simulated a normal distribution ($Y_{\text{normal}}$), a right skewed distribution ($Y_{\mathrm{rightskew}}$), and a left skewed distribution ($Y_{\mathrm{leftskew}}$) to have exactly mean 10.5 years and standard deviation 1.1 years. To visualize the right and left skewed distributions, we simulated 1,000,000 girls with age at attaining Tanner B2, both with a right skewed distribution ($Y_{\mathrm{rightskew}}$) and a left skewed distribution ($Y_{\mathrm{leftskew}}$). The right and left skewed distributions are shown as histograms in Supplementary Figure 4 and 5 and as cumulative incidence functions in Supplementary Figure 6 and 7, all compared against the normal distribution.

**2.2 Means, medians, standard deviations and 90-percentiles**

The mean and standard deviations were exactly 10.5 and 1.1 years for all three distributions. Further, the median and 90-percentile for the normal distribution were also exact. The medians and 90-percentiles of the two skewed distributions were obtained by simulating 10,000,000 individuals from each distribution ($Y_{\mathrm{rightskew}}$and $Y_{\mathrm{leftskew}}$) and calculating the medians and 90-percentiles (Supplementary Table 2).

**2.3 Simulation approach and performance measures**

In all simulations, we simulated the age at Tanner B2 for 8,000 girls (as this was the number of girls in our empirical data) and made the data left, right, and interval censored by simulating half-yearly replies to the questionnaires similar to the Puberty Cohort. Then, we derived performance measures, repeated the simulation in 10,000 replications and averaged the performance measures. Below is a more detailed description of how the half-yearly replies were simulated and how the performance measures were obtained. An overview of the three simulation experiments is shown in Supplementary Table 3.

We mimicked the age at replying to each questionnaire in the Puberty Cohort, and from these ages we made the observations either left, right and interval censored. To ensure 90% left-censored observations in simulation experiment 1 and 2, the age at replying to the first questionnaire was simulated by adding the 90-percentile (from Supplementary Table 2) and a random normal variable with mean 0 and standard deviation 0.0625 year. Then, we simulated the age at replying to subsequent questionnaires by adding the absolute value of a normal random variable with mean 0.5 year and a standard deviation of 0.0625 year to the age at the previous questionnaire. In total, we simulated 12 questionnaires, and thus the 12th questionnaire was simulated to be given at an age of around 17 years. To ensure around 60-65% interval-censored observations in simulation experiment 3, we simulated the age at replying to the first questionnaire at age 9.5 years plus a random normal variable with mean 0 and standard deviation 0.0625 years. We simulated further four subsequent half-yearly questionnaires as described for simulation experiment 1 and 2. If the simulated age at attaining Tanner B2 was between the ages of two questionnaires, this girl’s age at Tanner B2 was interval censored with an upper and a lower limit corresponding to the ages at which these two questionnaires were replied. If the age at Tanner B2 was less than the age at the first questionnaire, this girl’s age at Tanner B2 was left censored. If the age at Tanner B2 was later than the age at the last questionnaire, this girl’s age at Tanner B2 was right censored.

We used the interval censored regression model in STATA 15.1 MP software (Statacorp, College Station, TX) to estimate the age at attaining Tanner B2 in all three simulation experiments.^2^ We repeated this in S=10,000 simulations and extracted 1) the mean age at attaining Tanner B2 estimated using interval censored regression 2) the bias in median and mean age at Tanner B2, and 3) coverage of the nominal 95% confidence interval for the true mean and median value for age at Tanner B2 for each of the 10,000 simulations. Bias was defined as follows:

${Bias}_{\mathrm{mean}}= \frac{1}{S}\sum_{s=1}^{S} (\hat{\alpha}(s)-\theta_{\mathrm{mean}})$

And

${Bias}_{\mathrm{median}}= \frac{1}{S}\sum_{s=1}^{S} (\hat{\alpha}(s)-\theta_{\mathrm{median}})$

where $\hat{\alpha}(s)$ was the estimated mean age at Tanner B2 based on the normal distribution model for the *s*’th simulated data set, and $\theta_{\mathrm{median}}$ is the true median age at Tanner B2 and $\theta_{\mathrm{mean}}$ is the true mean age at Tanner B2. The coverage probability is computed as the fraction of nominal 95% confidence interval which captured the true mean or median (i.e., the true mean and median of the distributions we simulate from).

**3 RESULTS**

The results are shown in Supplementary Table 4. In simulation experiment 1 with 90% left censored observations of a normal distribution, the interval censored regression model was unbiased with nominal coverage. In simulation experiment 2 with 90% left censored observations of right and left skewed distributions, the results were biased with +/- 0.35 year (4.2 months) for the true mean and +/- 0.26 year (3.1 months) for the true median age at Tanner B2. In simulation experiment 3 with 60-65% interval-censored observations in the middle of right and left skewed distributions, the results were biased with +/- 0.05 year (0.6 month).

**4 COMMENT**

**4.1 Principal findings**

As expected, we found that our regression model was unbiased with nominal coverage with substantive left censoring as long as the assumption of the normal distribution was correct. In simulation experiment 2, we imposed realistic violations to the assumption of the normal distribution. The imposed skewness was in the same magnitude as the skewness observed for later milestones in the empirical data in the Puberty Cohort. In the case of substantive left-censoring and considerably skewed data, we found considerable bias of around 3-4 months. In simulation experiment 3, we had substantial interval censoring as well as some right and left censoring, but since observations were obtained over the middle of the age range of the distribution, bias was around half a month, which can be considered negligible.

**4.2 Interpretation**

Simulation results show that the estimated mean and median of the distribution are sensitive to departures from normality, when there is substantive censoring in one direction, for example left-censoring. Our results for Tanner Stage 2 should therefore be interpreted very cautiously as they depend on the validity of the normality assumption. By contrast, estimates are robust when there is substantial interval censoring. In this case we, so to speak, still retrieve sufficient information from the entire range of the distribution for estimates to maintain validity. For all milestones later than Tanner Stage 2, we have data on around 60% or more of the middle of the age distribution, and consequently, our estimates for all later pubertal milestones seem robust to considerable violations to the normal distribution.

**5 REFERENCES**

1. Fleishman AI. A method for simulating non-normal distributions. *Psychometrika*. 1978; 43:521-532.

2. Cameron AC, Trivedi PK. *Microeconometrics using Stata*. College Station, Tex.: Stata Press; 2010.
